# Development of Autoantibodies Following BNT162b2 mRNA COVID-19 Vaccination and Their Association with Disease Flares in Adult Patients with Autoimmune Inflammatory Rheumatic Diseases (AIIRD) and the General Population: Results of 1-Year Prospective Follow-Up Study

**DOI:** 10.3390/vaccines11020476

**Published:** 2023-02-17

**Authors:** Tal Gazitt, Tali Eviatar, Jacqueline Shear, Roni Meidan, Victoria Furer, Joy Feld, Amir Haddad, Muna Elias, Nizar Hijazi, Nili Stein, Pninit Shaked Mishan, Anna Zetser, Hagit Peleg, Ori Elkayam, Devy Zisman

**Affiliations:** 1 Carmel Medical Center, Rheumatology Unit, Haifa 3436212, Israel; 2 Division of Rheumatology, University of Washington Medical Center, Seattle, WA 98195-6428, USA; 3 Tel Aviv Medical Center, Rheumatology, Tel Aviv 6423906, Israel; 4 Sackler Faculty of Medicine, Tel Aviv University, Tel Aviv 69978, Israel; 5 The Ruth and Bruce Rappaport Faculty of Medicine, Technion, Haifa 31096, Israel; 6 Department of Community Medicine and Epidemiology, Carmel Medical Center, Haifa 3436212, Israel; 7 Microbiology and Immunology Laboratory, Carmel Medical Center, Haifa 3436212, Israel; 8 Rheumatology Unit, Hadassah Medical Center, Jerusalem 91120, Israel; 9 Hadassah Medical Center, Faculty of Medicine, Jerusalem 9112102, Israel

**Keywords:** mRNA vaccine, COVID-19, SARS-CoV-2, autoantibodies, disease flares

## Abstract

Development of autoantibodies following BNT162b2 mRNA COVID-19 vaccination and their association with disease flares in adult patients with autoimmune inflammatory rheumatic diseases (AIIRD) and the general population: results of 1-year prospective follow-up study. We conducted a prospective study aimed at investigating the incidence of appearance of autoantibodies (antinuclear, antiphospholipid, and rheumatoid factor) in the sera of 463 adult patients with AIIRD compared to 55 controls from the general population prior to, and following the second and third vaccine doses, and at 1-year of follow-up. Pre- and post-vaccination disease activity indices and the association of autoantibodies with rheumatic disease flares and new onset AIIRD were examined. Autoantibody development of any type in AIIRD patients vs. the controls was 4.0% (vs. 6.7%, *p* = 0.423) following two vaccine doses and 7.6% (vs. 0%, *p* = 0.152) after three doses. There was no significant difference in sex, age, or disease-type among individuals with and without autoantibody development, regardless of the immunosuppressant use. More patients developed autoantibodies following the third than the second vaccine dose (*p* = 0.004). Disease flares occurred in 5.8% and 7.2% of AIIRD patients following second and third vaccine doses, respectively, with autoantibody production increasing the risk of flares following the second (*p* = 0.002) and third (*p* = 0.004) vaccine doses. BNT162b2 vaccination resulted in the development of autoantibodies in a minority of AIIRD patients and controls. Autoantibody development was associated with disease flares in patients, but no new-onset autoimmunity was observed.

## 1. Introduction

Since the inception of the COVID-19 pandemic, and as previously demonstrated in other infections, COVID-19 has become associated with a wide range of clinical manifestations, including autoimmune features such as subacute thyroiditis, coagulopathy and antiphospholipid syndrome, immune thrombocytopenic purpura (ITP), autoimmune hemolytic anemia, and Guillain–Barré syndrome (GBS) [1,2]. Indeed, recent studies have shown that SARS-CoV-2-mediated autoantibody production occurs in a significant proportion of patients hospitalized with COVID-19 infection, including antiphospholipid antibodies (aPL) and antinuclear antibodies (ANA) [3,4]. While several reports have shown an association between SARS-CoV2-mediated autoantibody production and more severe clinical disease course in these patients [3,5,6], the long-term clinical implications of these autoantibodies are not known.

The SARS-CoV-2-provoked COVID-19 pandemic has fostered the development and authorization of novel messenger RNA (mRNA) vaccines that have since proven to be immunogenic and effective in the immunocompetent population [7,8]. Although patients with autoimmune inflammatory rheumatic diseases (AIIRD) were prioritized for COVID-19 vaccination [9], little information is currently available regarding the possible generation of autoantibodies following mRNA vaccination and their association with disease flares and/or clinically evident new-onset autoimmunity.

In order to address this knowledge gap, the objective of our study was to investigate the incidence of autoantibody development in a prospective cohort of AIIRD patients following three doses of BNT162b2 mRNA vaccination and their possible association with disease flares/new-onset autoimmunity in AIIRD patients in comparison to the general population.

## 2. Materials and Methods

### 2.1. Study Population

This study included 463 consecutive AIIRD patients recruited from Carmel and Tel Aviv Souraski Medical Centers as part of the prospective observational multicenter study investigating the immunogenicity and safety of two doses and a third (booster) dose of BNT162b2 mRNA COVID-19 vaccine in adult AIIRD patients compared to immunocompetent controls conducted between December 2020 and January 2022, as reported earlier by our group [10]. The study was performed in accordance with the principles of the Declaration of Helsinki and was approved by the research ethics committees (institutional review boards) of the participating medical centers: TLV-1055-20, CMC-0238-20, and HMO-0025-21. All study participants gave written informed consent upon recruitment into the study. All consenting adult (aged ≥18 years) AIIRD patients who fulfilled the ACR/EULAR criteria for rheumatoid arthritis (RA) [11], the CASPAR criteria for psoriatic arthritis (PsA) [12], the Assessment of SpondyloArthritis International Society (ASAS) classification criteria for ankylosing spondylitis (AS) [13], Systemic Lupus International Collaborating Clinics classification criteria for systemic lupus erythematosus (SLE) [14], classification criteria for systemic sclerosis (SSCL) [15], Chapel Hill classification criteria for systemic vasculitis (ANCA-associated vasculitis, giant cell arteritis, and other systemic vasculitides) [16], or the EULAR/ACR classification criteria for idiopathic inflammatory myopathy (IIM) [17] were recruited to this study. The control group was composed of 55 immunocompetent individuals, mostly healthcare workers. Exclusion criteria for all groups were pregnancy, history of past vaccination allergy, and lack of pre-existing serologic data on ANA, aPL, and rheumatoid factor (RF) for individuals with positive serologic results obtained for the first-time post-vaccination, and for controls—history of AIIRD and immunosuppressive treatment. Patients and controls with COVID-19 infection, confirmed by positive polymerase chain reaction (PCR) or antigen testing, were included in the study only if they became infected with COVID-19 after at least two mRNA vaccine doses with autoimmune serologic data available prior to COVID-19 infection and were subsequently excluded from the study once COVID-19 infection occurred, with data being censored from the time point of COVID-19 infection onward.

### 2.2. Data Collection

Demographic and clinical characteristics, including AIIRD diagnosis and disease-modifying anti-rheumatic drugs (DMARDs), including conventional DMARDs (cDMARDs), biologic DMARDs (bDMARDs) and targeted synthetic DMARDs (tsDMARDs), were reported by the participants and confirmed by reviewing the electronic medical records (EMRs) by the study investigators (TG, TE, JS, RM, VF, JF, AH, ME, and NH). The dates of administration of the BNT162b2 mRNA vaccines and dates of PCR or antigen testing for COVID-19 infection, when relevant, were recorded.

### 2.3. Vaccination Procedure

All participants were administered the two-dose regimen of the BNT162b2 Pfizer BioNTech mRNA vaccine according to national protocol guidelines, namely, each 30 μg dose was given as an intramuscular injection in the deltoid muscle, with the second dose given 3 weeks following the first dose. A third, or booster dose, was given at least 5 months later according to the national immunization protocol.

### 2.4. Vaccine Immunogenicity

BNT162b2 mRNA vaccine immunogenicity was evaluated by measuring the serum IgG antibody levels against SARS-CoV-2 trimeric spike S1/S2 glycoproteins using the LIAISON (DiaSorin) quantitative binding assay, performed at 2–6 weeks following the second vaccine dose. This food and drug administration-authorized assay has a clinical sensitivity and specificity above 98%. A value above 15 binding antibody units (BAUs) was considered positive, according to the manufacturer’s instructions [18].

### 2.5. Vaccine-Related Autoantibody Development

We compared the development of autoantibodies (ANA, aPL, and RF) in the sera of AIIRD patients (≥18 years) compared to general population controls prior to, and 2–6 weeks following, the second and the third (booster) vaccine doses as well as at 1-year of follow-up from initial vaccination. In this study, ANA was measured by immunofluorescence (IF), which was read manually by a laboratory technician and by multiplex bead assay (ANA Screen, BIO-RAD) for detection of anti-dsDNA, anti-Smith, anti-smRNP, anti-RNP, anti-Scl-70, anti-chromatin, anti-ribosomal P, and anti-Jo-1 antibodies as well as anti-SSA and anti-SSB antibodies. The IF cutoff was set at 1:160 dilution, which was in accordance with clinical practice. Anti-dsDNA titer was validated by the nDNA Crithidia Luciliae (^®^AESKUSLIDES). Serologic testing for RF was performed using the immunoturbidimetric assay cobas c701 (Roche Pharmaceuticals, Ltd, Basel, Switzerland) with cutoff of >15 units for positivity. All serologic testing of patient samples from both medical centers was analyzed at one center (Carmel Medical Center).

Data on the development of autoantibodies were analyzed based on the number of patients with available serology following each vaccine dose. When a baseline time-point was missing prior to obtaining mRNA COVID-19 immunization, the baseline pre-vaccination autoantibody was imputed to be negative only if negative serologic testing results for that particular autoantibody was observed following vaccination. Patients with initial, positive post-vaccination serologic testing without previous, pre-vaccination serologic testing results were excluded from the study. Post-vaccination seroconversion was defined as any first-time ANA, aPL, or RF seropositivity in patients with available pre-vaccination tests.

### 2.6. Vaccine Safety and Assessment of AIIRD Activity

The study participants were queried (by telephone or in-person) about adverse events 2 weeks after the first vaccine dose, with an in-person visit 2–6 weeks following the second vaccine dose and 2–6 weeks following the third vaccine dose to assess for adverse events and AIIRD activity. Adverse events were reported when they occurred at the injection site or when they occurred in temporal proximity to vaccination.

Information on pre-vaccination disease activity during the 3 months preceding vaccination was retrieved from the medical records. Post-vaccination disease activity was clinically assessed 2–6 weeks after the second and third vaccine doses and at the 1-year endpoint. Pre- and post-vaccination disease activity indices and clinical signs/symptoms of disease flare or new-onset disease were assessed as appropriate for each AIIRD: Clinical Disease Activity Index (CDAI), Simplified DAI (SDAI), and DAS-28-CRP for rheumatoid arthritis (RA); Disease Activity in Psoriatic Arthritis (DAPSA), Leeds Enthesitis and Dactylitis Indices, Psoriasis Area Severity Index (PASI) for psoriatic arthritis (PsA); Bath Ankylosing Spondylitis Disease Activity Index (BASDAI) and Ankylosing Spondylitis Disease Activity Score (ASDAS) for ankylosing spondylitis (AS); Systemic Lupus Erythematosus Disease Activity Index (SLEDAI) for SLE; Modified Rodnan Skin Score (mRSS) for SSCL; and Patient and Physician Global Assessment (PGA and PhGA, respectively), by using a visual analogue scale (VAS) of 0–10 mm for SSCL, vasculitis, and IIM. Suspicion of post-vaccination disease flares given an increase in disease activity score was verified by two independent assessors (JS and RM) examining clinical visit notes, comprising of disease activity scores, and the treating rheumatologist’s clinical impression, with consensus needed between the reviewers to decide upon actual occurrence of disease flare. Post-vaccination increase in disease activity was attributed to vaccination if the patient had discontinued DMARD therapy ≤1 month from the time of vaccination with subsequent disease flare occurrence.

### 2.7. Statistical Analysis

Continuous variables are summarized as mean ± standard deviation (SD) while categorical variables are presented as numbers and proportions. Comparisons of baseline characteristics between patients with AIIRD and controls were performed using Chi square test or Fisher’s exact test, as appropriate. Continuous variables were compared using independent *t*-test. McNemar test was used to evaluate changes in developing autoantibodies after the initial two vaccine doses and the development of autoantibodies after the third vaccine dose. All data were analyzed using SPSS, 28 (IBM Corp. Released 2022. IBM SPSS Statistics for Windows, version 28.0, 2022, Armonk, NY, USA). In all analyses, *p* ≤ 0.05 for the 2-tailed tests was considered statistically significant.

## 3. Results

### 3.1. Study Participants

This prospective study included 463 AIIRD patients and 55 controls. Out of the 686 AIIRD patients initially recruited to the study, 223 patients were excluded for the following reasons: 212 lacked prior serologic data in all 3 autoantibody categories, 2 had COVID-19 infection prior to obtaining at least two vaccine doses with associated serologic testing, 2 did not complete at least two vaccine doses, and 7 were lost to follow-up. Out of the initially recruited 59 controls, 4 were lost to follow-up. Of the AIIRD patients, 9 were included only until the completion of the first and second vaccine doses with associated post-vaccination serologic testing and were then excluded from the rest of the study due to COVID-19 infection.

Female patients constituted 72.8% (n = 337) of patients and 69.1% (n = 38) of the controls (*p* = 0.562) (Table 1). Compared to the controls, the patients were significantly older (mean age of patients 58.1 ± 14.6 vs. 52.0 ± 14.4 in controls, (*p* = 0.003). AIIRD patients included RA (37.6%), PsA (22.0%), and SLE (12.1%)), with two SLE patients having had kidney transplants, vasculitis (9.7%), AS (9.1%), SSCL (5.4%), and myositis (3.7%), with 56.2% (n = 260) treated with biologic therapy. The majority of both patients and controls received a third vaccine dose ((99.5% (n = 405) vs. (98.2%, n = 54), respectively), although the third dose vaccination data were missing for 11.9% (n = 55) in AIIRD patient group.

### 3.2. Autoantibody Development

The development of any autoantibody-type based on the available serologic data from 453 AIIRD patients vs. 55 controls following two vaccine doses was 3.9% (n = 18) vs. 6.7% (n = 3) (*p* = 0.423), with a single AIIRD patient developing both ANA and aPL seropositivity. Autoantibody development of any type following three vaccine doses based on the available data from 355 AIIRD patients was 7.7% (n = 31) vs. 0% (n = 0) (*p* = 0.152), with the same AIIRD patient found to have persistent dual ANA and aPL seropositivity. The two individuals with SLE who had also undergone kidney transplants did not develop autoantibodies during the study.

When examining specific subtypes of autoantibodies formed by AIIRD patients following two vaccine doses, a new ANA seropositivity (ANA titer of >1:160 dilution) was found in 3.2% (n = 15) (missing data on 0.4% (n = 2) patients); a new aPL seropositivity was found in 0.4% (n = 2) of patients (missing data on 2.2% (n = 10) patients); and a new RF seropositivity (RF > 15 units) was found in 0.4% (n = 2) patients (missing data on 0.2% (n = 2) patients). Following three vaccine doses, 4.5% (n = 16) of patients developed ANA seropositivity, 1.1% (n = 4) of patients developed aPL seropositivity, and 3.4% (n = 12) of patients developed RF seropositivity.

The persistence of autoantibodies at the end of the study following three vaccine doses for those who seroconverted in the initial round of the post-vaccination testing was found in two out of fifteen cases of ANA seroconversion (end-of-study serologic testing was absent for four patients and another patient was excluded from the study midway due to COVID-19 infection); in two out of two cases of aPL seroconversion (data includes the individual with dual ANA and aPL seroconversion); and in none out of two cases of RF seroconversion.

Notably, there was no significant difference in sex, age, or disease-type among AIIRD patients who developed autoantibodies vs. those who did not, following two (Table 2a) and three (Table 2b) vaccine doses. Autoantibody development of any type was also not found to be associated with any particular immunosuppressant use or class of immunosuppressants (cDMARDs, bDMARDs, or tsDMARDs), and neither was there any significant difference in autoantibody development when comparing between TNFα antagonists and anti-B-cell-directed therapy following two or three vaccine doses (*p* = 0.30 vs. *p* = 0.51, respectively). Moreover, autoantibody development was also not found to be associated with a measured immunologic response to BNT162b2 vaccine following the second vaccine dose, although more patients developed autoantibodies following the third than the second vaccine dose (n = 30 patients of 355 patients with available serologic data who also obtained a third vaccine dose vs. n = 18 patients of 453 patients with available serologic data who obtained two vaccine doses, *p* = 0.004). Using the McNemar test on the available serologic data collected from 323 patients, there was a specifically higher risk in the development of RF seropositivity following the third than the second vaccine dose (*p* = 0.013), which was not found for ANA (*p* = 0.263) or aPL (*p* = 0.500).

In the immunocompetent control group, ANA seroconversion occurred in three cases, with no individuals undergoing aPL or RF seroconversion. There was no statistically significant difference in the autoantibody development between AIIRD patients vs. the controls following two (*p* = 0.481) and three (*p* = 0.094) vaccine doses.

### 3.3. Disease Flares

Disease flares among AIIRD patients occurred in a minority of patients from the available data (5.8%, n = 27 of 423 patients following two vaccine doses and 7.2%, n = 29 of 212 patients following three vaccine doses), with autoantibody production increasing the risk of flares following the second and third vaccine doses (*p* = 0.002 vs. *p* = 0.004, respectively). Indeed, 5/17 (29.4%) of AIIRD patients with autoantibody production flared following the second vaccine dose, and 9/30 (30.0%) of AIIRD patients with autoantibody production flared following the third vaccine dose. Disease flares were not found to be associated with a measured immunologic response to the BNT162b2 vaccine following the second or third vaccine doses (*p* = 0.097 vs. *p* = 0.255, respectively). Neither were they found to be associated with any specific AIIRD disease-type, even when subdividing the AIIRD-types into three groups consisting of inflammatory arthritis, connective tissue diseases, and vasculitis (*p* = 0.51 vs. *p* = 0.26 following two and three vaccine doses, respectively). Notably, no signs of new-onset inflammatory or autoimmune disease were reported in study participants, regardless of the autoantibody development.

## 4. Discussion

In our prospective, 1-year study analyzing the development of autoantibodies following two and three doses of the BNT162b2 mRNA COVID-19 vaccination, we found that vaccination resulted in the development of mostly transient autoantibodies in a minority of AIIRD patients and controls, with no added risk for autoantibody formation among AIIRD patients relative to immunocompetent individuals. Autoantibody development increased the risk of flares. Notably, after a 1-year follow-up, no new-onset autoimmune disease was observed in our study participants, regardless of the autoantibody formation.

The pioneering use of mRNA vaccines in clinical practice has raised concern for the potential formation of autoantibodies and associated autoimmune phenomena. This concern stems from several observations. First, previous research has demonstrated autoantibody formation following various non-mRNA vaccinations in healthy subjects, such as Toplak et al. [19], who demonstrated the de novo formation or increase in ANA and aPL antibody titers in up to 15% of participants of a cohort of 92 healthy adult subjects post-seasonal influenza vaccination, with 8% showing a persistence of autoantibodies. Reassuringly, however, no associated autoimmune phenomena were noted by the authors. Similarly, a study on aPL antibody formation in healthy volunteers after recombinant hepatitis B vaccination [20] in AIIRD patients and healthy volunteers following seasonal influenza and/or H1NI vaccines [21] noted a mostly transient elevation in aPL titers in a minority of subjects. Examined in light of this historic data on autoantibody formation following non-mRNA vaccines, our data on low incidence of mostly transient autoantibody formation are reassuring, in that the safety profile of mRNA vaccines in this regard is comparable to that of other vaccine types.

A second source of concern in utilizing mRNA vaccines, irrespective of post-vaccination autoantibody formation, is that of new-onset autoimmunity, given previous reports of new-onset autoimmunity following non-mRNA vaccines, such as human papillomavirus, hepatitis B, and influenza vaccines, possibly triggered by molecular mimicry [22,23]. Specific concerns in the case of mRNA vaccines lies in their mechanism of action, in that the mRNA/DNA vaccine adjuvant works by engaging Toll-Like-Receptors (TLRs) 7/8 and TLR9, and thus can lead to the TLR7-dependent hyper-stimulation of age-associated CD11c+ T-bet+ B cells (ABCs) that are implicated in the production of autoreactive immunoglobulin G, the enhanced presentation of antigens to T cells, and the spontaneous formation of germinal centers [24,25]. These ABCs, which can accumulate prematurely in autoimmune conditions, such as SLE, and generate autoreactive antibody-secreting plasmablasts in genetically and immunologically predisposed individuals, can lead to autoimmunity [24]. The activation of TLR7 and TLR9 can in turn lead to the production of interferon I, which is a key cytokine in the development of SLE and other rheumatologic diseases [26].

In the case of non-mRNA vaccines, examples of such reported post-vaccination autoimmunity include Guillain–Barré Syndrome (GBS) as the most frequently reported autoimmune neurological adverse event following seasonal influenza vaccination in the Vaccine Adverse Event Reporting System (VAERS) [27], or Henoch–Schonlein purpura, with the induction of aPL antibodies in the reported pediatric cases following influenza vaccination [28,29,30]. Specifically in the case of mRNA COVID-19 vaccines, a growing body of evidence associates these vaccinations with several autoimmune conditions, including myocarditis [31], immune thrombocytopenic purpura [22,32], and immune-medicated myositis [33]. As in previous non-mRNA vaccinations, the number of cases of reported autoimmune manifestations following COVID-19 are small, and whether the association between COVID-19 vaccines and autoimmune manifestations is coincidental or causal remains to be elucidated. For instance, the 68,000 cases of post-COVID-19 vaccination GBS, occurring during the four-billion-person immunization program conducted over a 1-year period did not exceed those expected to occur naturally, regardless of any immunization plan [22]. In other cases, such as in the study on post-vaccination myocarditis by Mevorach et al., where the incidence of autoimmune phenomena was higher than the expected incidence based on historical data (standardized incidence ratio of 5.34), cases were still rare, limited to male recipients between the ages of 16 and 19 years, and were mild in severity [31]. In reviewing the historical and current reporting of post-immunization autoimmunity, the majority of published epidemiological studies to date have not confirmed causality between vaccinations and autoimmune diseases, especially given that most AIIRD occur in the absence of immunizations, the incidence of post-vaccination autoimmune phenomena are rare (estimated to occur at <1/10,000 vaccine doses), and the voluntary reporting systems of vaccine-related adverse events, such as VAERS, which require the filing of reports on any suspicion of adverse event irrespective of the degree of certainty regarding actual vaccine causality [34]. Additionally, in contrast to the randomization design of pre-vaccine licensure trials, post-licensure epidemiologic studies are ‘observational’ in nature, which means that the allocation to the immunized and unimmunized populations are no longer random, with a higher risk of inadequate control for biases and confounding factors [34]. Our data show that no novel post-immunization autoimmune phenomena were observed in both AIIRD patients and healthy controls in our cohort, which is reassuring.

In our review of the literature, we found only a single study regarding autoantibody formation in AIIRD patients post-mRNA COVID-19 vaccination by Blank et al., which, reassuringly and similarly to our study, reported low incidences of ANA seroconversion in both the patients with inflammatory arthritis and the controls (i.e., 11.4% vs. 7.7%, respectively, *p* = 0.731) [35]. As in our study, autoantibody formation was unrelated to the immunologic response to mRNA vaccination (19). Moreover, as in our study, autoantibody levels were typically of a low titer and were transient, in contrast with autoantibody formation following COVID-19 infection, with data showing at least a single autoantibody that was present in approximately 50% of hospitalized patients [4]. Consistently, Thurm et al. also reported a transient increase in autoantibodies that are commonly associated with SLE, RA, celiac disease, antiphospholipid syndrome post-homologous and heterologous COVID-19 mRNA/vector vaccination in healthy individuals with pre-existing autoantibodies relative to healthy individuals without pre-existing autoantibodies, with none of the healthy volunteers developing clinical signs of autoimmune disease [36]. Along these lines, a study by Noureldine Ha et al. on ANA and aPL formation in healthy individuals following the initial and second dose of the BNT162b2 vaccine showed no association between BNT162b2 vaccine administration and changes in aPL and ANA titers [37]. Similar results were also demonstrated in yet another post-vaccination study on 354 healthy subjects by Ṥwierkot et al. In this study, a possible correlation between post-vaccination ANA titer and the severity of vaccine adverse events was noted, though no autoimmunity developed de novo following vaccination [38].

In comparison to our study, Blank et al. noted a higher self-reported disease flare incidence following vaccination (27.7% overall disease flare incidence found by Blank et al. vs. 5.8% and 7.2% following second and third vaccine doses in our study, respectively), with our study results being more in line with the 4.4% post-mRNA vaccination flare incidences reported in a large real-world study supported by the European League Against Rheumatism (EULAR), including 5121 AIIRD patients [39], as well as the 11.0% flare incidence reported in a smaller study of post-mRNA vaccination in 1377 AIIRD patients by Connolly et al. [40], and 11.4% post-mRNA vaccination flares in a study on SLE patients [41]. This difference may stem from the reliance by Blank et al. on virtual patient visits and patient reporting of disease flares, whereas we had in-person clinic visits with actual joint counts and disease activity score assessments noted both by patients and physicians with disease activity scores verified by an independent assessor. Additional notable differences between our study design and that of Blank et al., which may have contributed to variability in results, was our focus on a single type of mRNA vaccine for all patients and controls, while Blank et al. used two different mRNA vaccines—BNT162b2 and mRNA-1273 in AIIRD patients, and BNT162b2 in controls. Moreover, in the study by Blank et al., 18.8% of arthritis patients and 11.5% of immunocompetent controls had a previous COVID-19 infection, which in itself is known to be associated with autoantibody formation, and may have also affected the autoantibody formation and associated disease flares in their study [4].

Interestingly, unlike our study, autoantibody formation in the study by Blank et al. was not associated with an increase in inflammatory arthritis flares. These difference in findings may stem from differences in study cohorts: our study included a larger AIIRD cohort (463 vs. 138 patients) and was not limited to inflammatory arthritis, but also included patients with connective tissue diseases and vasculitis. In addition, our study included three doses of mRNA vaccination in the majority of AIIRD patients and controls vs. two vaccine doses in the study by Blank et al.

The limitations of our study include the small number of cases with autoantibody formation among AIIRD patients and controls, making it difficult to draw definite conclusions on the risk factors related to autoantibody development and persistence as well as any definite association with disease flares requiring treatment changes. The strengths of our study include the large number of patients with a large variety of rheumatologic conditions with corresponding data on immunologic response of these individuals to the vaccination and extensive serologic testing including ANA by IF, multiplex, and Crithidia assays. In addition, this study represents data on a 1-year follow-up of three doses of the mRNA vaccine with in-person visit assessments of disease flares with chart verification by independent assessors.

## 5. Conclusions

In summary, in our prospective cohort of AIIRD patients and controls, incidences of autoantibody seroconversion after three doses of mRNA COVID-19 vaccination was low, with many of these autoantibodies appearing to be transient. Importantly, no new-onset autoimmune disease was observed in study participants, regardless of autoantibody formation after 1-year of follow-up. Overall, these study results support the safety of the mRNA COVID-19 vaccination in AIIRD patients, with minimal concern for subsequent autoimmune manifestations.

## Figures and Tables

**Table 1:** Baseline characteristics of the study population.

		AIIRD Patients (n = 463)	Controls (n = 55)	*p*-Value
**Age (Mean ± SD)**		58.1 ± 14.6	52.0 ± 14.4	0.003
**Sex (Female) N, %**		72.8% (n = 337)	69.1% (n = 38)	0.562
**Diagnosis**	**RA**	37.6% (n = 174)	N/A	
	**PsA**	22.0% (n = 102)	N/A	
	**AS**	9.1% (n = 42)	N/A	
	**SLE**	12.1% (n = 56)	N/A	
	**SSCL**	5.4% (n = 25)	N/A	
	**Vasculitis**	9.7% (n = 45)	N/A	
	**IIM**	3.7% (n = 17)	N/A	
	**SS**	0.2% (n = 1)	N/A	
	**IGG4-RD**	0.2% (n = 1)	N/A	
**AIIRD Medications**	**NSAIDs**	2.2% (n = 10)	N/A	
	**GCS**	18.8% (n = 87)	N/A	
	**HCQ**	17.7% (n = 82)	N/A	
	**MTX**	28.3% (n = 131)	N/A	
	**SSZ**	2.2% (n = 10)	N/A	
	**LEF**	4.5% (n = 21)	N/A	
	**AZA**	3.9% (n = 18)	N/A	
	**MMF**	5.0% (n = 23)	N/A	
	**ANTI-TNF**	23.1% (n = 107)	N/A	
	**ANTI-IL-6**	5.8% (n = 27)	N/A	
	**ANTI-IL-23**	0.9% (n = 4)	N/A	
	**ANTI-IL17**	5.8% (n = 27)	N/A	
	ANTI-CD20 *	16.0% (n = 74)	N/A	
	**BEL**	1.9% (n = 9)	N/A	
	**ABT**	2.6% (n = 12)	N/A	
	**PDE4**	0.9% (n = 4)	N/A	
	**JAK-I**	6.5% (n = 30)	N/A	
	**IVIG**	1.5% (n = 7)	N/A	
	**COLCHICINE**	1.1% (n = 5)	N/A	
**DMARDs by category**	**cDMARDs**	38.9% (n = 180)	N/A	
	**bDMARDs**	56.2% (n = 260)	N/A	
	**tsDMARDs**	7.3% (n = 34)	N/A	
**Third vaccination**	**Yes**	99.5% (n = 405 **)	98.2% (n = 54)	0.317
**Any new autoantibody positivity after second vaccine dose**	**Yes**	4.0% (n = 18/453 with available data)	5.6% (n = 3/54 with available data)	0.481
**Any new autoantibody positivity after third vaccine dose**	**Yes**	8.7% (n = 31/355 with available data)	0% (n = 0/32 with available data)	0.094

Abbreviations: ABT = abatacept, AIIRD = autoimmune rheumatic disease, anti-IL = anti-interleukin, anti-TNF = anti-tumor necrosis alpha, SSCL: classification criteria for systemic sclerosis, AS = ankylosing spondylitis, AZA = azathioprine, BEL = belimumab, DMARDs = disease-modifying anti-rheumatic drugs, GCS = glucocorticosteroids, HCQ = hydroxychloroquine, IGG4-RD = immonoglobulin G4-related disease, IIM = inflammatory myositis, JAK-I = janus kinase inhibitors, LEF = leflunomide, MMF = mycophenolate mofetil, MTX = methotrexate, n = number, NSAIDs = non-steroidal anti-inflammatory drugs, PsA = psoriatic arthritis, RA = rheumatoid arthritis, SD = standard deviation, SLE = systemic lupus erythematosus, SS = Sjogren’s syndrome, SSZ = sulfasalazine. * Patients on anti-CD20 therapy received their BNT162b2 vaccinations 5 months following their last infusion and 1 month prior to their next infusion. ** A total of 407 patients had data regarding their 3rd vaccine dose.

**Table 2:** a: Comparison between autoantibody-positive AIIRD patients to AIIRD patients without autoantibody production following second vaccine dose. b: Comparison between autoantibody-positive AIIRD patients to AIIRD patients without autoantibody production following third vaccine dose.

(a)				
		Any Autoantibody Positivity	No Autoantibody Positivity	*p*-Value
**Number of patients with serologic data**	n = 453	4.0% (n = 18)	96.0% (n = 435)	
**Age** **(Mean ±** **SD)**		57.8 ± 14.5	58.0 ± 14.6	0.942
**Sex**	**Female**	4.9% (n = 16)	95.1% (n = 312)	0.176
	**Male**	1.6% (n = 2)	98.4% (n = 123)	
**Diagnosis**	**RA**	3.6% (n = 6)	96.4% (n = 161)	0.308
	**PsA**	4.0% (n = 4)	96.0% (n = 97)	
	**SLE**	9.3% (n = 5)	90.7% (n = 51)	
	**AS**	7.1% (n = 3)	92.9% (n = 39)	
	**Vasculitis**	0.0% (n = 0)	100% (n = 45)	
	**SSCL**	0.0% (n = 0)	100% (n = 23)	
	**Myositis**	0.0% (n = 0)	100% (n = 17)	
	**SS**	0.0% (n = 0)	100% (n = 1)	
	**IgG4-RD**	0.0% (n = 0)	100% (n = 1)	
**Vaccine immune response (seroconversion)**	**Yes**	4.7% (n = 16 of 338)	95.3% (n = 322 of 338)	0.492
**DMARDs**	**cDMARDs**	4.7% (n = 8)	95.3% (n = 162)	0.536
	**bDMARDs**	3.9% (n = 10)	96.1% (n = 246)	0.933
	**tsDMARDs**	5.9% (n = 2)	94.1% (n = 32)	0.637
**(b)**				
		**Any Autoantibody Positivity**	**No Autoantibody Positivity**	***p*** **-Value**
**Number of patients with serologic data**	n = 355	7.7% (n = 31)	80.0% (n = 324)	
**Age** **(Mean ± SD)**		60.2 ± 13.7	58.4 ± 14.6	0.506
**Sex**	**Female**	9.6% (n = 25)	90.4% (n = 236)	0.347
	**Male**	6.4% (n = 6)	93.6% (n = 88)	
**Diagnosis**	**RA**	12.0% (n = 16)	88.0% (n = 117)	0.604
	**PsA**	6.3% (n = 5)	93.8% (n = 75)	
	**SLE**	4.9% (n = 2)	95.1% (n = 40)	
	**AS**	6.9% (n = 2)	93.1% (n = 27)	
	**Vasculitis**	10.8% (n = 4)	89.2% (n = 33)	
	**SSCL**	0.0% (n = 0)	100% (n = 17)	
	**Myositis**	13.3% (n = 2)	86.7% (n = 13)	
	**SS**	0.0% (n = 0)	100% (n = 1)	
	**IgG4-RD**	0.0% (n = 0)	100% (n = 1)	
**Vaccine immune response (seroconversion)**	**Yes**	9.3% (n = 26 of 279)	90.7% (n = 253 of 279)	>0.99
**DMARDs**	**cDMARDs**	11.5% (n = 15)	88.5% (n = 116)	0.165
	**bDMARDs**	7.5% (n = 15)	92.5% (n = 185)	0.350
	**tsDMARDs**	0.0% (n = 0)	100% (n = 27)	0.150

Abbreviations: AS = ankylosing spondylitis, DMARDs = disease-modifying anti-rheumatic drugs, IGG4-RD = immonoglobulin G4-related disease, SSCL: classification criteria for systemic sclerosis, n = number, PsA = psoriatic arthritis, RA = rheumatoid arthritis, SD = standard deviation, SLE = systemic lupus erythematosus, and SS = Sjogren’s syndrome.

## Data Availability

The data used and/or analyzed during the present study are available from the corresponding author on reasonable request.
